# The Efficacy of Expiratory Muscle Training during Inspiratory Load in Healthy Adult Males: A Randomized Controlled Trial

**DOI:** 10.3390/healthcare10050933

**Published:** 2022-05-18

**Authors:** Toshiya Tsukamoto, Michitaka Kato, Yasunari Kurita, Masaki Uchida, Akira Kubo, Hitoshi Maruyama

**Affiliations:** 1 Department of Shizuoka Physical Therapy, Faculty of Health Science, Tokoha University, 1-30 Mizuochi, Aoi-ku, Shizuoka 420-0831, Japan; katomanzooo@sz.tokoha-u.ac.jp (M.K.); ykurita@sz.tokoha-u.ac.jp (Y.K.); m.uchida@sz.tokoha-u.ac.jp (M.U.); 2 Anti-Aging Center, Ginza Hospital, 4-12-15 Ginza, Chuo-ku, Tokyo 104-0061, Japan; ak@kuboakira.com; 3 Graduate School, International University of Health and Welfare, 4-1-26 Akasaka, Minato-ku, Tokyo 107-8402, Japan; hmaru@iuhw.ac.jp

**Keywords:** expiratory muscle training, respiratory muscle fatigue, maximum inspiratory mouth pressure, maximum expiratory mouth pressure

## Abstract

This study aimed to evaluate the effects of expiratory muscle training (EMT) on respiratory muscle strength and respiratory distress during inspiratory load. Thirty-one healthy adult males were randomly divided into an EMT group who underwent EMT (*n* = 15) and a control group who did not undergo EMT (*n* = 16). The EMT group underwent EMT with a 50% load of maximum expiratory mouth pressure (PEmax) for 15 min, twice a day, every day, for 4 weeks. The parameter of respiratory muscle fatigue was a decrease in maximum inspiratory mouth pressure (PImax) and PEmax during 20 min of inspiratory load; thus, PImax and PEmax during inspiratory load were measured. Respiratory distress during inspiratory load was assessed using the Borg scale. These assessments were performed on the same subjects in each group before and after the 4 week study. In the EMT group, the PEmax values after the study were significantly higher than those before the study (*p* < 0.01). Furthermore, before the study, the PImax and PEmax values for the EMT group during inspiratory load were significantly lower than those before inspiratory load (*p* < 0.01). However, after the study, there was no difference in these values between during and before inspiratory load. In the EMT group, the Borg scale value during inspiratory load from 6 to 20 min was significantly lower after the study than before the study (*p* < 0.05). EMT increases expiratory muscle strength, thereby attenuating decreased respiratory muscle strength (PImax and PEmax) and respiratory distress during inspiratory load in healthy subjects.

## 1. Introduction

Respiratory muscle strength decreases in patients with chronic obstructive pulmonary disease (COPD) [1]; therefore, they use a higher percentage of maximum inspiratory mouth pressure (PImax). This is closely associated with respiratory distress [2] and induces respiratory muscle fatigue [3].

Exertional dyspnea restricts activities of daily living (ADL) in patients with COPD and impairs their health-related quality of life (QOL) [4]. The basic pathophysiology of exertional dyspnea includes airflow restriction due to peripheral airway obstruction and dynamic hyperinflation [5]. Peripheral airway obstruction and dynamic hyperinflation lead to respiratory muscle fatigue. Reduced respiratory muscle contractile force associated with respiratory muscle fatigue is also related to reduced exercise tolerance by promoting dyspnea [6]. In addition, reduced respiratory muscle strength associated with respiratory muscle fatigue is related to increased mortality in patients with COPD [7].

Respiratory rehabilitation, which has been established as a non-drug therapy for COPD, reportedly reduces the rate of readmission and mortality in patients with COPD while improving health-related QOL [8]. Respiratory muscle training, an element of respiratory rehabilitation, is employed as a treatment for respiratory muscle fatigue in COPD [9]. Particularly, inspiratory muscle training (IMT) contributes to not only relieving dyspnea but also improving inspiratory muscle strength, exercise tolerance, and health-related QOL [3,10].

Alternatively, respiratory muscle fatigue occurs in not only inspiratory muscles but also expiratory muscles [11,12]. Particularly, fatigue of the abdominal muscles, which are expiratory muscles, affects the occurrence of respiratory muscle fatigue in healthy subjects [12]; thus, expiratory muscle training (EMT) may be effective in preventing respiratory muscle fatigue. However, few studies have reported the relationship between EMT and respiratory muscle fatigue.

A device with resistive threshold loading is most commonly used for respiratory muscle training [13]. This device contains a spring- and weight-loaded valve, and developed pressure opens the valve. Such features allow the threshold load to be set independent of the flow rate. A PImax of ≥30% increases inspiratory muscle strength when this device is used for IMT [3]. Based on a previous study evaluating EMT [14], the present study performed EMT with a 50% load of maximum expiratory mouth pressure (PEmax).

The expiratory muscle in patients with COPD is activated during expiration [15]. Expiratory muscle strength is associated with airflow obstruction and dynamic hyperinflation [16]. EMT increases expiratory muscle strength, improves abdominal muscle tone, and attenuates dynamic hyperinflation, possibly resulting in the elevation of the diaphragm [14]. Based on these findings, we hypothesized that EMT increases respiratory muscle strength and fatigue endurance of the respiratory muscles, thereby reducing the occurrence of respiratory muscle fatigue caused by inspiratory load.

A previous study has shown that inspiratory load is associated with fatigue in the inspiratory and expiratory muscles and increased abdominal pressure caused by the expiratory muscle affects diaphragm contraction [12]. EMT is a useful tool for improving both inspiratory and expiratory muscle strength [17]. Based on these reports, we believe that EMT suppresses fatigue in the inspiratory and expiratory muscles caused by inspiratory load.

This study was performed to investigate the effects of 4 weeks EMT on respiratory muscle fatigue.

## 2. Methods

This study was approved by the Ethics Review Committee of the International University of Health and Welfare (16-Io-140). All subjects received a detailed explanation of the study before providing their written consent. This study was conducted in compliance with the Consolidated Standards of Reporting Trials guidelines [18].

### 2.1. Subjects

The subjects were recruited via a bulletin board at Tokoha University between January and April 2017. The inclusion criteria were males aged 20–39 years. The exclusion criteria were individuals with a history of smoking, respiratory disease, neurological disease, and history of orthopedic disease of the neck and trunk. The study participants were graduates of Tokoha University. The subjects consisted of 31 healthy adult males, who were then randomly divided into two groups—the EMT group who underwent EMT for 4 weeks (*n* = 15) and the normal control (NC) group who did not undergo EMT (*n* = 16).

To examine respiratory muscle fatigue, inspiratory load should be performed at baseline (i.e., absence of fatigue in the respiratory muscle) and changes in muscle recovery should be observed over time [19]. Patients with COPD may experience decreased respiratory muscle strength and respiratory muscle fatigue due to airway obstruction and dynamic hyperinflation. Thus, evaluation of the effects of training methods on respiratory muscle fatigue is difficult. In this study, healthy subjects were evaluated for respiratory muscle fatigue at baseline (i.e., absence of fatigue), during inspiratory load, and after resting.

### 2.2. Research Protocol and EMT

The design of this study was a randomized controlled trial (RCT). In terms of the randomization method, the subjects were divided in a ratio of 1:1 using a random number table by simple randomization. The subjects were enrolled and allocated into groups by the researchers.

Respiratory muscle fatigue and respiratory distress during inspiratory load as well as respiratory functions at rest of the subjects in the EMT and NC groups were assessed before the study for a baseline. The EMT group underwent EMT using EMST150^®^ (Aspire Products, LLC, Cape Carteret, NC, USA). In terms of the intervention protocol for the EMT group, the subjects underwent EMT for 15 min, twice a day, every day, for 4 weeks using an expiratory load of 50% of maximum expiratory mouth pressure (50% PEmax) [14,20,21]. Except for the addition of EMT, the subjects in the EMT group continued with the same ADL as before the study and noted their EMT on record sheets. The subjects in the NC group also continued with their ADL as before the study throughout the period of 4 weeks.

After the study period, the measurements were repeated.

### 2.3. Measurement Items

#### 2.3.1. Respiratory Muscle Fatigue

The main outcome of this study was respiratory muscle fatigue. The Respiratory Muscle Fatigue Workshop Group [19] has defined respiratory muscle fatigue as “a state of reduced contractile force or contractile rate of active muscles due to loads, which recovers by resting the muscles.” Thus, this study used the decrease in the maximum inspiratory mouth pressure (PImax) and maximum expiratory mouth pressure (PEmax) values, which were measured during the inspiratory load, as indicators of respiratory muscle fatigue [12,22].

For respiratory muscle strength, PImax and PEmax were measured by connecting a respiratory muscle strength meter unit (respiratory muscle strength meter ASS, Minato Medical Science Corporation) to a spirometer (Autospiro AS-507, Minato Medical Science Corporation). For limb positioning, the measurement was performed with a nose clip attached while the patient sat in a chair and held the respiratory muscle strength meter using the left upper limb. The method of Black and Hyatt [23] was adopted for the measurement of respiratory muscle strength, where maximum inspiration and expiration were measured three times, and the maximum values were represented as PImax and PEmax. The measurements were performed and the equipment were used according to the standard method published in the American Thoracic Society (ATS)/European Respiratory Society (ERS) statement [24].

#### 2.3.2. Respiratory Distress

The 10-level modified Borg scale (Borg scale) [25] was used as the indicator of respiratory distress.

Inspiratory Load and Respiratory Muscle Fatigue Assessment Procedure.

**Figure 1:**
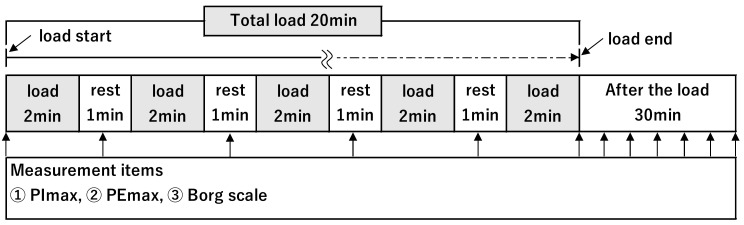
Procedure for inspiratory load and assessment of respiratory muscle fatigue. Borg scale, modified Borg scale; EMT, expiratory muscle training group; NC, normal control group; PImax, maximum inspiratory mouth pressure; PEmax, maximum expiratory mouth pressure.

Figure 1 depicts the inspiratory load procedure and the measurement procedure for respiratory muscle fatigue. The study by Tsukamoto et al. [12] was referred to for the measurement procedure. First, PImax and PEmax were measured before applying inspiratory load. Furthermore, 50% of PImax measured before the inspiratory load (50% PImax) was adopted as the inspiratory load pressure. Threshold IMT^®^ (Respironics New Jersey Inc., Parsippany, NJ, USA) or POWERbreathe PLUS^®^ (POWERbreathe International Ltd. Southam, Warwickshire, UK) was used as the inspiratory load equipment. The subjects performed inspiratory load for 2 min and then rested for 1 min; these 3 min constituted one set. The subjects repeated the process for 10 sets, and the total time of inspiratory load was 20 min. There were 15 breaths per minute during the inspiratory load, and the inspiratory and expiratory times were 2 s each. The PImax, PEmax, and Borg scale were measured during each resting period of 1 min. After the inspiratory load ended, PImax, PEmax, and Borg scale were measured every 5 min.

#### 2.3.3. Respiratory Functions

For respiratory functions, forced vital capacity (FVC), percentage forced vital capacity (%FVC), forced expiratory volume in 1 s (FEV_1.0_), forced expiratory volume % in 1 s (FEV_1.0%_), and peak expiratory flow (PEF) at rest were measured three times each using a spirometer, and the maximum value of each parameter was adopted. The respiratory function test was performed according to the ATS standard method [20].

### 2.4. Sample Size

The sample size was calculated with a power of 0.80 and a significance level of 0.05 using the PEmax values, as reported in a previous study [12]. EZR, version1.51, was used as the statistical software (http://www.jichi.ac.jp/saitama-sct/SaitamaHP.files/statmedEN.html) (accessed on 10 August 2020). Thus, the sample size of each group was calculated to be 15, with a total of 30 subjects.

### 2.5. Statistical Analysis

Paired *t*-test was performed to compare respiratory muscle strength and respiratory functions before and after the study for both groups. Two-way repeated-measures factorial analysis of variance (group and measurement time) was performed to assess the changes in PImax and PEmax during the inspiratory load in both groups. When interaction was confirmed, Bonferroni method was used as a post hoc test. Wilcoxon signed-rank test was performed to compare the Borg scale scores before and after the study. The data were presented as the mean ± standard deviation for PImax and PEmax and as the median (25th and 75th percentiles) for Borg scale scores. *p*-values of <0.05 were used to denote statistical significance. JSTAT, version 13.0, was used for statistical processing.

## 3. Results

**Figure 2:**
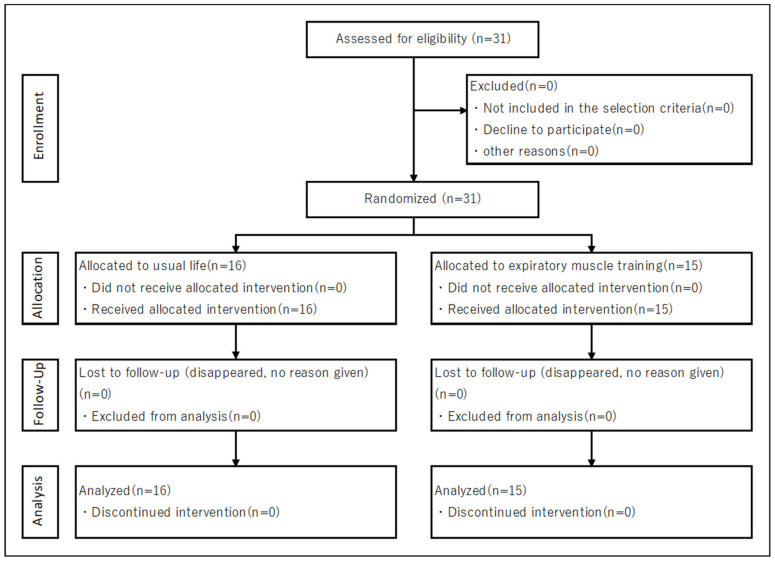
The CONSORT flow diagram.

Figure 2 shows the flow diagram of this study. All subjects were included in the analysis, and there were no dropouts throughout the study period. In addition, the EMT implementation rate in the EMT group was 97.4%.

Table 1 shows the subject characteristics, respiratory functions, and respiratory muscle strength before and after the study in both groups. In the EMT group, the PEmax and PEF values after the study were significantly higher than those before the study (*p* < 0.01 and *p* < 0.05). In both groups, no significant difference in other items was found before and after the study.

**Figure 3:**
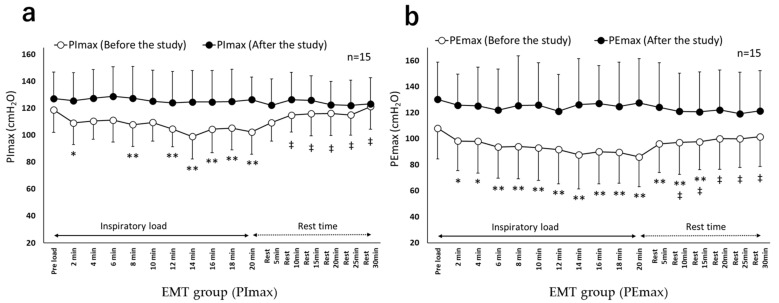
Chronological changes in PImax and PEmax (EMT group). (**a**). Chronological changes in PImax (EMT group). (**b**). Chronological changes in PEmax (EMT group). EMT group: expiratory muscle training group; PImax: maximum inspiratory mouth pressure; PEmax: maximum expiratory mouth pressure. Unit, cmH_2_O, mean ± standard deviation. Compared with pre-load within group, * *p* < 0.05 and ** *p* < 0.01; compared with after-load within group (20 min), ^‡^ *p* < 0.01.

**Figure 4:**
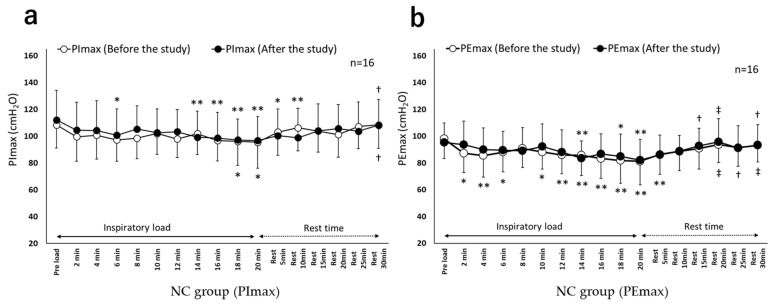
Chronological changes in PImax and PEmax (NC group). (**a**). Chronological changes in PImax (NC group). (**b**). Chronological changes in PEmax (NC group). NC group: normal control group; PImax: maximum inspiratory mouth pressure; PEmax: maximum expiratory mouth pressure. Unit, cmH_2_O, mean ± standard deviation. Compared with pre-load within group, * *p* < 0.05 and ** *p* < 0.01; compared with after-load within group (20 min), ^†^ *p* < 0.05, ^‡^ *p* < 0.01.

Figure 3 and Figure 4 show the chronological changes in the PImax and PEmax values during the inspiratory load before and after the study in both groups. For the PImax and PEmax values, a significant interaction was observed between the groups and the measurement time (*p* < 0.01 for both). In the EMT group, before the study, the PImax and PEmax values were significantly lower during and after the inspiratory load than those before the load (*p* < 0.01). However, after the study, no difference in the PImax and PEmax values was observed between during the inspiratory load and after the inspiratory load when compared to before the inspiratory load.

**Figure 5:**
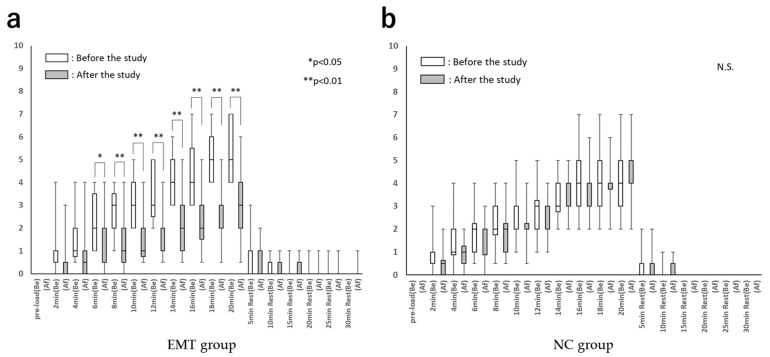
Chronological changes in the Borg scale. (**a**). Chronological changes in the Borg scale (EMT group). (**b**). Chronological changes in the Borg scale (NC group). Borg scale: modified Borg scale; EMT group: expiratory muscle training group; NC group: normal control group; Be: before the study; Af: after the study. Compared with before the study within each group, * *p* < 0.05 and ** *p* < 0.01. N.S.: not significant.

Figure 5 shows the chronological changes in the Borg scale scores before and after the study in both groups. In the EMT group, after the study, the Borg scale scores during the inspiratory load were significantly lower than those before the study (*p* < 0.01).

## 4. Discussion

This study was conducted to verify the effects of 4 week EMT on respiratory muscle fatigue. The results clarified that EMT increases PEmax and reduces respiratory muscle fatigue during the inspiratory load when compared with those before the study. In addition, it was determined that EMT significantly reduces respiratory distress during inspiratory load. To the best of our knowledge, this is the first RCT to clarify the effects of EMT on respiratory muscle fatigue.

In terms of respiratory muscle strength, the PEmax values in the EMT group increased significantly, reaching 22.1 cmH_2_O (approximately 20%) after the study compared with those before the study. Mota et al. [14] have implemented a 5 week EMT with a load of 50% PEmax in patients with COPD and reported that PEmax increased by 19%. In addition, Neves et al. [17] have reported an increase of 21.5 cmH_2_O in PEmax using the same methods, and the increase in PEmax in this study is considered similar to that in previous studies. The subjects of this study were healthy adult males. However, the effects conform to those reported in previous studies involving patients with COPD [14,17]. In addition, no significant difference in PImax was found before and after the study in the EMT group; however, an increase of 8.3 cmH_2_O (approximately 7%) was observed after the study compared with PImax before the study. The reason is unclear, but it has been reported that the activities of abdominal muscles enhance the activity efficiency of inspiratory muscles [26]; thus, the increase in expiratory muscle strength might have affected the activation of inspiratory muscles.

In terms of respiratory functions, in the EMT group, PEF significantly increased after the study compared with that before the study. Kojima et al. [27] have reported that EMT strengthens the expiratory muscles, making them effective in improving peak cough flow (PCF), an indicator of coughing ability. In addition, positive correlations between PEmax and PCF [28] and between PCF and PEF have been reported [29]. Therefore, the increase in expiratory muscles by EMT is considered to be strongly related to the increase in PEF observed in this study.

In terms of respiratory muscle fatigue, in the EMT group, before the study, the PImax and PEmax values decreased significantly during the inspiratory load compared with those before the inspiratory load, which recovered after the inspiratory load. Respiratory muscle fatigue has been defined as “a state of reduced contractile force or contractile rate of active muscles due to loads, which recovers by resting the muscles” [19]. Thus, in the EMT group, respiratory muscle fatigue might have occurred in both inspiratory and expiratory muscles due to the inspiratory load before the study. Alternatively, in the EMT group, after the study, PImax and PEmax did not decrease significantly during the inspiratory load, asserting the ability of EMT to reduce respiratory muscle fatigue. A study on respiratory muscle fatigue and EMT has reported that the significant increase in expiratory muscle strength by EMT is associated with improved exercise tolerance, suggesting reduced oxygen consumption of the respiratory muscles when active [14]. Ramirez-Sarmiento et al. [30] have reported that IMT increases both type I and type II respiratory muscle fibers and augments inspiratory muscle strength as well as endurance. Based on these findings, it was estimated that in the EMT group in this study, respiratory muscle fatigue decreased due to the decrease in oxygen consumption and anaerobic metabolism of respiratory muscles during continuous application of a constant load. A meta-analysis [3] has not clarified the effects of IMT in reducing respiratory muscle fatigue. Thus, EMT may be effective in improving and preventing respiratory muscle fatigue in patients with COPD by reducing respiratory muscle fatigue in both inspiratory and expiratory muscles.

In terms of the respiratory distress and respiratory muscle fatigue, Borg scale scores during inspiratory load in the EMT group were significantly lower after the study than those before the study, suggesting that EMT reduces respiratory distress during inspiratory load.

Killian et al. [31] have reported that when inspiratory load is applied, respiratory distress is considered a respiratory effort sensation. This sensation may be due to the requirement of a relatively large respiratory output because of the decrease in maximum muscle strength under circumstances in which the respiratory muscle strength is decreased (motor command theory) [32,33]. Thus, EMT reduces the relative respiratory effort by increasing the respiratory muscle strength and decreasing the respiratory distress.

This study showed that EMT increased the expiratory muscle strength and attenuated respiratory muscle fatigue and respiratory distress caused by inspiratory load. Respiratory muscle fatigue causes both inspiratory and expiratory muscle fatigue, but EMT inhibits both. However, because this study included only healthy adult males, immediately applying the results of this study to COPD patients who experience decreased respiratory muscle strength, respiratory distress, and dynamic hyperinflation may be difficult. Thus, using the results of this study as the basic document, several types of evaluations involving patients with COPD should be performed. Specifically, we believe that the efficacy of EMT, load intensity of EMT, and efficacy of EMT when combined with IMT and different types of exercise methods should be continuously examined. Although this study examined only healthy adult males, periodic evaluation of respiratory muscle strength in patients with COPD is important for maintaining function of this muscle, which leads to the successful management of respiratory distress and attenuation of respiratory muscle fatigue.

This study has several limitations. First, allocation concealment could not be achieved as the subjects were allocated by the researchers. Second, since the assigned subjects, interventionists, and outcome assessors were not blinded, information bias might have occurred. Third, as the subjects were healthy adult males, it is unclear as to whether the results from this study can be extrapolated to patients with COPD. Fourth, lung volume was not specified when inspiratory load was performed. Females were excluded from the target population with the aim of decreasing the risk of hyperventilation syndrome. Fifth, the sample size was small. Further studies with a higher number of patients are necessary. Finally, to prevent glottis closure and buccinator use, air leaks occurred in the measurement circuit during the measurement of respiratory muscle strength in accordance with ATS/ERS [24]. This value is small and not of concern, but measuring the mouth pressure that regulates lung capacity may be necessary. Hence, further investigations taking these points into consideration are needed.

## 5. Conclusions

This study proved that in healthy subjects, EMT reduces respiratory muscle fatigue in both inspiratory and expiratory muscles during the inspiratory load by increasing the expiratory muscle strength. Furthermore, it was shown that the increase in respiratory muscle strength by EMT lowers the relative respiratory effort and alleviates respiratory distress.

## Figures and Tables

**Table 1:** Effects of EMT on subject characteristics, respiratory function, and respiratory muscle strength.

	EMT *n* = 15		NC *n* = 16	
	Before the Study	After the Study	Before the Study	After the Study
Male sex, *n* (%)	15 (100)	-	16 (100)	-
Age, years	27.3 ± 2.4	-	26.7 ± 5.1	-
Height, cm	173.2 ± 5.0	-	170.5 ± 5.4	-
Body weight, kg	64.8 ± 7.4	64.5 ± 7.3	67.0 ± 7.0	66.6 ± 6.9
Body mass index, kg/m^2^	21.5 ± 1.5	21.5 ± 1.8	23.1 ± 2.1	22.9 ± 2.1
Respiratory function				
FVC, L	4.5 ± 0.5	4.6 ± 0.6	4.4 ± 0.4	4.4 ± 0.4
%FVC, %	106.3 ± 10.7	107.3 ± 11.6	104.5 ± 9.8	104.6 ± 10.3
FEV1.0, L	4.0 ± 0.4	4.0 ± 0.4	3.8 ± 0.4	3.8 ± 0.4
FEV1.0_%_, %	88.2 ± 5.9	87.8 ± 6.2	87.1 ± 4.2	86.7 ± 3.7
PEF, L/s	9.5 ± 1.1	10.2 ± 1.1 *	9.8 ± 1.1	9.6 ± 0.9
Respiratory muscle strength				
PImax, cmH_2_O	118.8 ± 16.8	127.1 ± 19.7	108.4 ± 17.3	112.1 ± 22.1
PEmax, cmH_2_O	108.0 ± 23.7	130.1 ± 28.6 **	98.1 ± 14.8	95.3 ± 14.5

EMT, expiratory muscle training group; NC, normal control group; FVC, forced vital capacity; %FVC, percentage forced vital capacity; FEV1.0, forced expiratory volume in one second; FEV1.0%, forced expiratory volume % in one second; PEF, peak expiratory flow; PImax, maximum inspiratory mouth pressure; PEmax, maximum expiratory mouth pressure. Mean ± standard deviation, compared with before the study within each group, * *p* < 0.05, ** *p* < 0.01.

## Data Availability

Please contact the authors for data requests.
